# Dynamics of Serologic Change to Gluten in Celiac Disease Patients

**DOI:** 10.3390/nu15245083

**Published:** 2023-12-12

**Authors:** Jack Syage, Ana Ramos, Vasiliy Loskutov, Anna Norum, Adam Bledsoe, Rok Seon Choung, Matthew Dickason, Jennifer Sealey-Voyksner, Joseph Murray

**Affiliations:** 1 ImmunogenX, Inc., 1600 Dove Street, Suite 330, Newport Beach, CA 92660, USA; aramos@immunogenx.com (A.R.); vloskutov@immunogenx.com (V.L.); mdickason@immunogenx.com (M.D.);; 2 Division of Gastroenterology and Hepatology, Mayo Clinic, Rochester, MN 55905, USA

**Keywords:** celiac disease, serology, gluten, gluten-free diet (GFD), tTG-IgA

## Abstract

Serologic measures of tissue transglutaminase (tTG) immunoglobulin A (IgA) and deamidated gliadin peptide (DGP) IgA and immunoglobulin G (IgG) are hallmark tests utilized when diagnosing individuals for celiac disease (CeD) and for monitoring adherence to a gluten-free diet (GFD), currently the only available treatment for CeD. We address two issues in this study: (i) the relapse to seropositivity for CeD patients who resume a gluten containing diet and (ii) the correlation between two different tTG-IgA assays near the upper limit of normal (ULN) designated thresholds. Regarding the first issue, often a suspected CeD individual is put back on a gluten diet to return to their serologic levels. However, we show it requires a substantial amount of gluten for serology to return to a positive level. For example, in one study of 22 patients treated with placebo and taking 84 g of gluten over 6 weeks, only two converted from seronegative to seropositive for tTG-IgA. Regarding the second topic, we compare the relationship for different serologic assays, namely tTG-IgA AB (recombinant, ULN = 4 units/mL) vs. tTG-IgA (non-recombinant, ULN = 20 units). There is a strong correlation between both measurements as evidenced by a Pearson coefficient of R = 0.8584; however, we observed that the cross-correlation in terms of sensitivity and specificity improved substantially by using an ULN value of three instead of four for the tTG-IgA AB (recombinant) assay. This result suggests that assay thresholds used for initial diagnosis in patients who have not yet started a GFD may need to be adjusted for monitoring and in the setting of a diagnostic gluten challenge.

## 1. Introduction

Celiac disease (CeD) is a chronic disorder of the small intestine, often genetically linked, triggered by gluten exposure [1,2]. It’s prevalent in roughly 1% of most populations [3]. The only current treatment is a strict, lifelong gluten-free diet. This diet, while effective in lessening symptoms and intestinal damage, is challenging for many patients to maintain and may lack vital nutrients [4]. CeD primarily affects the small intestine’s proximal epithelium, causing villous atrophy as a result of an immune reaction to wheat, rye, or barley. Even minimal gluten exposure can perpetuate inflammation, heightening risks of serious health issues like lymphoma, bowel cancer, osteoporosis, anemia, and malnutrition [5,6]. About half of those with CeD experience moderate to severe symptoms, leading to significant financial and personal strain on themselves, their families, and friends [7].

Serology tests are commonly used to screen for celiac disease (CeD), an autoimmune disorder triggered by the ingestion of gluten in genetically susceptible individuals [8,9,10]. These tests measure certain antibodies in the blood that are associated with the disease. tTG-IgA assays are most used in the diagnosis and treatment of CeD. The test is generally performed using an enzyme-linked immunosorbent assay (ELISA). It has been reported that the tTG-IgA test has a sensitivity of 78% to 100% and a specificity of 90% to 100% for diagnosing CeD in individuals with a gluten-containing diet [11]. However, these results can be dependent on the degree of intestinal damage and the gluten intake of the suspected individual. The DGP tests are not considered as sensitive as tTG-IgA [12] but are useful in circumstances such as those of young children who may not be as sensitive to tTG-IgA [13,14]. In a recent study on children at risk of CeD, it was observed that of a proportion of children who registered tTG-IgA seroconversion that 74% showed an earlier increase in DGP IgA readings [4]. Another study provided a meta-analysis for comparative results of tTG-IgA and DGP-IgA for children <2 years of age showing that, although tTG-IgA provides better sensitivity and specificity, the addition of DGP-IgA results increased the overall accuracy of diagnosing this infant population [14]. Serology is also a useful way to select CeD patients for clinical trials where effectiveness of new treatments may be dependent on gluten exposure of the patient [15,16,17,18,19,20].

The connection between serologic titer readings and gluten intake, whether unintentionally or in a gluten challenge clinical trial, is not immediate but can significantly lag a change in gluten intake. A particular issue is when suspected CeD individuals have already self-administered a GFD and therefore may register negative in serologic tests. Although the patient could still then be prescribed to have a biopsy, this is not always the case for a negative serology reading. Often the individual is put back on a gluten diet to restore their serologic levels. The aim of this work is to present evidence showing that a serologic change in gluten may be slower and lower than commonly assumed, and relying on restoring a gluten diet in suspected CeD patients to reestablish a positive serologic titer may be fallacious.

Another topic presented in this manuscript is a comparison of two different versions of tTG-IgA assays to determine the strength of the correlation, particularly in the upper limit of normal (ULN) diagnostic threshold region in treated patients suspected of exposure to gluten.

## 2. Materials and Methods

The test results reported here are from two recent celiac disease trials, IMGX003-NCCIH-1721 (NCT03585478) [21] and IMGX003-NIAID-1821 (NCT04243551). In both trials, serum was collected and analyzed by enzyme-linked immunosorbent assay (ELISA) for antibodies tTG-IgA (QUANTA Lite^®^ R h-tTG IgA (recombinant) and QUANTA Lite^®^ h-tTG IgA (non-recombinant)) and DGP-IgA, DGP-IgG (QUANTA Lite^TM^ Gliadin IgA II, QUANTA Lite^TM^ Gliadin IgG II), all assays were by INOVA Diagnostics, Inc., San Diego, CA. The IMGX003-NCCIH-1721 study samples were collected and analyzed at the Mayo Clinic. The IMGX003-NIAID-1821 study samples were collected at each of seven clinical trial sites and serum samples were sent for analyses to ACM Global Laboratories (QUANTA Lite, h-tTG IgA (non-recombinant), Gliadin IgA II, and Gliadin IgG II) and Quest Diagnostics (QUANTA Lite^®^ R h-tTG IgA (recombinant)).

Serologic change from baseline following a 6-week, 2 g per day gluten challenge study (IMGX003-NCCIH-1721) was evaluated for *n* = 22 patients receiving gluten plus placebo for the assays identified above. In this study, adult patients (18–80 years) were required to have physician-diagnosed, biopsy-confirmed CeD; to be following a GFD for a minimum of 12 months; and to have histologically well-controlled disease, as evidenced by a measured ratio of villus height to crypt depth Vh:Cd of ≥2.0.

The correlation of tTG-IgA (recombinant) and tTG-IgA (non-recombinant) was evaluated based on an on-going non-gluten challenge study (IMGX003-NIAID-1821) for a total of 347 paired titer readings (694 total readings). In this study, similar to the above study, adult patients (18–80 years) were required to have physician-diagnosed, biopsy-confirmed CeD; to be following a GFD for a minimum of 12 months; however, histology as represented by Vh:Cd was not measured or controlled. In this on-going trial, this currently represents 304 individual patients at initial screening for seroactivity (positive readings for any of tTG-IgA AB, DGP-IgA, or DGP-IgG). Of these screened patients, 83 were seroactive and, of these patients, 35 passed subsequent screening requirements and were randomized for treatment providing a second set of individual serologic readings. A final set of serology readings was conducted at completion of the treatment period and, at the time of locking the data for the analyses for this manuscript, that included 23 completed patients in this on-going trial. 

Statistical analyses were performed using GraphPad Prism 9.1.1 or XLSTAT 2021.1.1. In all cases the lower and upper readable thresholds of <1 and >100 were converted to 0 and 125 titer units to limit the full range of excessive values. For the tTG-IgA comparison a Pearson’s correlation test was performed to determine the correlation between the two immunoassay variants. Descriptive statistics were determined.

## 3. Results

### 3.1. Serologic Change Dynamics

**Figure 1:**
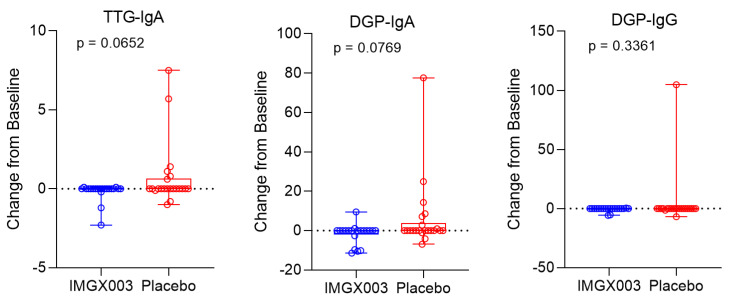
Serology results shown by box and whisker plots showing min and max, first and third quartile and median and ANCOVA *p*-value. Change from baseline refers to serology measurements before and after the 6-week gluten challenge period.

**Figure 2:**
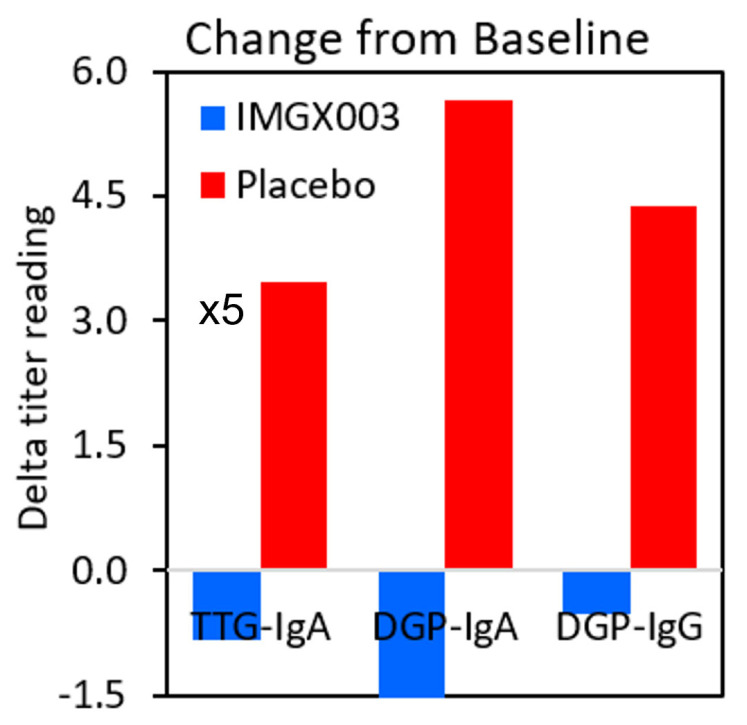
Mean change from baseline for all three titers. The tTG-IgA threshold for positivity is 4 units versus 20 units for DGP-IgA and DGP-IgG. We therefore multiplied the values by five in order to place all the results on the same scale.

Figure 1 shows the serologic changes from baseline for *n* = 22 enrolled patients in the IMGX003-NCCIH-1721 gluten-challenge study. After consuming 2 g of gluten daily for 6 weeks, a total of 84 g of gluten, no more than 6 out of 22 patients (≤23%) registered a positive change for any of the three measured titers (6 of 22 for tTG-IgA, 6 of 22 for DGP-IgA, and 1 of 22 for DGP-IgG), and only two patients went from seronegative to seropositive (for tTG-IgA). This strongly suggests that a regimen of taking gluten to reestablish seropositivity to diagnose individuals for celiac disease may require much greater quantities than previously believed. Figure 2 shows the mean change for the placebo group in the IMGX003-NCCIH-1721 study. These mean changes are well below the ULN values for tTG-IgA (ULN = 4 units/mL) and for DGP IgA and IgG (ULN = 20 units). As an interesting aside, the IMGX003 (active) group on average improved their serologic scores despite the gluten challenge [21].

### 3.2. Correlation of tTG IgA vs. tTG IgA AB Assays

**Figure 3:**
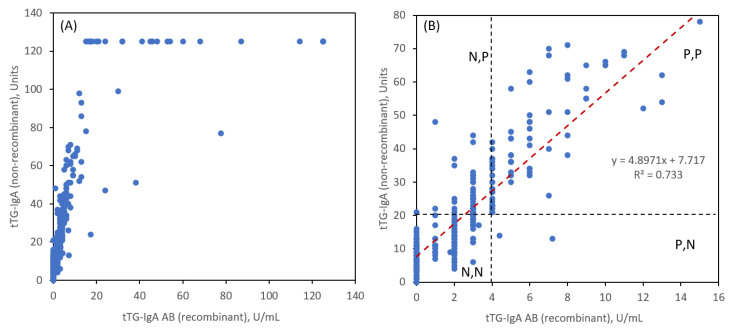
Correlation of titer readings for tTG-IgA AB (recombinant) and tTG-IgA (non-recombinant) assays: (**A**) All data, (**B**) expanded view with dotted lines representing the upper limit of normal (ULN) for each assay.

Recently a new tTG-IgA chemiluminescence assay (QUANTA Lite^®^ h-tTG IgA (non-recombinant)) was introduced with a ULN of 20 units matching that of currently used DGP-IgA and DGP-IgG assays. We compare this assay in paired readings of the same patient serum samples against the older tTG-IgA AB (recombinant) assay to assess the correlation and accuracy in the ULN threshold regions. Figure 3 shows the 347 paired readings. General linearity (Figure 3A) is observed for the two tTG-IgA assays. An expanded view (Figure 3B) shows the linear correlation near the ULN region along with the ULN thresholds for each assay. There is a strong correlation between both measurements, as evidenced by a Pearson coefficient R = 0.8584.

The notation N and P is for negative and positive readings based on <ULN and ≥ULN, respectively for tTG-IgA (AB = recombinant) and tTG-IgA (non-recombinant), respectively. It is evident that for a tTG-IgA AB ULN value of 4 units/mL, there are a significant number of readings that are in the N,P category (48 instances) and very few in the P,N category (two instances). Table 1 tabulates the number of such readings in addition to the more desirable agreements for N,N and P,P. To achieve better correlation at the ULN thresholds, we reduced the tTG-IgA AB ULN value to 3 units/mL and observed a much more balanced agreement for N,P (14 instances) and P,N (16 instances). If we define a combination sensitivity (sens) as the sum of N,N and P,P results as a percentage of total patient reads and 1-specificity (1-spec) by the sum of N,P and P,N we obtained the values in Table 1. These are not true sensitivity and specificity values since we have not compared the pairing of these two tTG-IgA assays to a controlled group of non-CeD patients, but they are instructive for evaluating the correlation and for suggesting refinements in the threshold ULN to be used for making diagnoses.

## 4. Discussion

In this study we address two issues regarding serologic immunoassay measurements for CeD: (i) the reestablishment of seropositivity for CeD patients who resume a gluten-containing diet and (ii) the correlation between two different tTG-IgA assays near the ULN designated thresholds.

Regarding the first topic, it is assumed that individuals who are suspected of having CeD and have already self-treated with a GFD can regain positive serology with a reasonable gluten regimen. There is very little in the literature indicating the appropriateness of this practice, yet it is commonly practiced. There is also impetus to develop diagnostic methodologies, in particular serologic assays, that can avert the need for biopsies, particularly in children.

Guidelines published by the European Society for Paediatric Gastroenterology Hepatology and Nutrition (ESPGHAN) [22] and by the American Gastroenterological Association recommend preliminary screening of adolescences by tTG-IgA [12]. If symptomatic patients register titer readings >10 ULN of tTG-IgA, the guidelines recommend further blood sampling to measure endomysial antibodies as an alternative to duodenal biopsies. Positive outcomes for these measures are sufficient to provide a CeD diagnosis in children provided their symptoms improve on a GFD. A key consideration, which is a primary topic of this study, is if the patient has already started a GFD resulting in a negative tTG-IgA, then the recommendation is to revert to a diet to include three slices of wheat bread daily for 1–3 months. Assuming a slice of bread contains about 4 g of gluten, this regimen accounts for about 100–300 g over a 1–3 month recommended period, although shorter periods of time can be justified [10]. We refer the reader to a review of other diagnostic techniques for CeD [23].

We showed that 84 g of gluten, consumed over a 6-week period, is inadequate for regaining seropositivity for tTG-IgA or DGP-IgA and DGP-IgG. Only 2 of 22 patients under this gluten challenge regimen reverted to a seropositive tTG-IgA response at 1× ULN in contrast to the 10× ULN response necessary to affirm a CeD diagnosis absent a biopsy determination (Figure 1).

Another gluten challenge study observed, among other measures, serologic changes from baseline [24] for gluten intake generally greater than the above study. For ‘moderate’ daily gluten intake, ranging from 3.3 to 5.0 g for days ranging from 29 to 91 (a total gluten intake ranging from 145 to 412 g), 5 out of 10 patients registered significant positive tTG-IgA seroconversion. For ‘low’ daily gluten intake, ranging from 1.3 to 2.8 g for days ranging from 77 to 103 (a total gluten intake ranging from 101 to 249 g), 4 out of 11 patients registered significant positive tTG-IgA seroconversion. Not surprisingly, these higher total gluten consumptions showed a higher proportion of seroconversion, but it is important to note from a diagnostic perspective, that a large percentage of these gluten-challenged patients would have been classified as CeD negative on this basis alone.

Other studies have addressed the uncertainty regarding the accuracy of diagnostic indicators for CeD following a GFD. In one such study, 20 patients with biopsy-confirmed CeD were subjected to 5.7 g of daily gluten for 14 days equating to a total gluten intake of 80 g [25], comparable to the IMGX003-NCCIH-1721 gluten challenge study of 84 g over 42 days [21]. This work did not report serology, but concluded that such gluten intake was not sufficient to establish significant mucosal architectural change observing a Vh:Cd reduction of about 0.4, consistent with the IMGX003-NCCIH-1721 study. However, a greater than 100% increase was observed for CD4^+^ effector-memory gut-homing HLA-DQ:gluten tetramer-binding T cells in blood in 12 of 15 evaluated participants, concluding that a larger study should investigate this biomarker as a potentially more sensitive assay for CeD.

Other potentially sensitive assays for CeD requiring less gluten intake have been reported. A new assay, based on measuring interleukin-2 (IL-2) levels following a single gluten dose, showed evidence as an immunological biomarker response for diagnosing for CeD [26,27,28]. In this case, an individual is given a short duration gluten challenge and whole blood is collected and incubated for a few hours and analyzed. Elevated levels of IL-2 are indicative of individuals with CeD and differentiate them from non-celiac gluten sensitivity [28]. This assay requires validation but could obviate the need to rely on an extended gluten challenge and diagnosis by tTG-IgA assays. Another alternative way is to use a novel approach, which is utilizing the neoepitopes derived from tTG-DGP complexes. Indeed, a recent study using these neoepitopes showed the promising accuracy to differentiate the mucosal healing in treated patients with CeD [29]. 

We now focus on the second topic of this study regarding the relative agreement of the two different tTG-IgA assays reported above. There are few such comparisons [30,31,32]. Other than reasonable agreement in the diagnostic threshold region near the ULN, an important insight revealed that perhaps the older tTG-IgA AB (recombinant) version should lower the ULN from a value of four to three. Supporting this premise is a very recent meta-analysis of a large population of diagnosed CeD patients, which reported that a significant proportion who were originally diagnosed in the upper normal tTG-IgA range were subsequently diagnosed to have CeD [33]. The threshold for positivity for patients with a normal gluten-containing diet may not provide reliable results compared to the context of a gluten challenge where the degree of and direction of change in CeD serology may be more sensitive. A persistently negative serological test after a period of gluten challenge may provide a false sense of security in ruling out CeD with potentially long-term implications for the patient. Although this revision may not have major impact on 1× ULN diagnostic information, it could be very impactful for singular diagnostic decisions based on the use of 10× ULN to circumvent for example biopsy confirmation in children, particularly in terms of negative predictive value of a higher value [34].

## Figures and Tables

**Table 1:** Negative and Positive Correlated Outcomes for tTG-IgA AB vs. tTG-IgA.

ULN tTG-IgA	N,N	P,P	N,P	P,N	Sens	1-Spec
≥4	192	105	48	2	86%	14%
≥3	178	139	14	16	91%	9%

N is negative <ULN, P is positive ≥ULN for tTG-IgA AB and tTG-IgA, respectively.

## Data Availability

Data may be made available upon request to the corresponding author.
